# The risk of post-polypectomy bleeding among patients receiving antithrombotic agents: A prospective observational study

**DOI:** 10.1590/1516-3180.2020.0305.R1.10122020

**Published:** 2021-03-22

**Authors:** Hilmi Bozkurt, Özlem Zeliha Sert, Tolga Ölmez, Zeynep Zehra Keklikkıran, Orhan Uzun, Selçuk Gülmez, Erdal Polat, Mustafa Duman

**Affiliations:** I MD, MSc. General Surgeon, Gastrointestinal Surgeon and Molecular Oncology Doctoral Student, University of Health Sciences, Haseki Research and Education Hospital, Istanbul, Turkey.; II MD, MSc. General Surgeon, Gastrointestinal Surgeon and Molecular Oncology Doctoral Student, University of Health Sciences, Haydarpaşa Research and Education Hospital, Istanbul, Turkey.; III MD. General Surgeon and Gastrointestinal Surgeon, University of Health Sciences, Koşuyolu Research and Education Hospital, Istanbul, Turkey.; IV MD. General Surgeon and Gastrointestinal Surgeon, University of Health Sciences, Koşuyolu Research and Education Hospital, Istanbul, Turkey.; V MD. General Surgeon and Gastrointestinal Surgeon, University of Health Sciences, Koşuyolu Research and Education Hospital, Istanbul, Turkey.; VI MD. General Surgeon and Gastrointestinal Surgeon, University of Health Sciences, Koşuyolu Research and Education Hospital, Istanbul, Turkey.; VII MD. Associate Professor, General Surgeon and Gastrointestinal Surgeon, University of Health Sciences, Koşuyolu Research and Education Hospital, Istanbul, Turkey.; VIII MD. Professor, General Surgeon and Gastrointestinal Surgeon, University of Health Sciences, Koşuyolu Research and Education Hospital, Istanbul, Turkey.

**Keywords:** Colonoscopy, Hemorrhage, Colonic polyps, Polypectomy, Colonoscopic polypectomy, Antithrombotic medication, Postpolypectomy bleeding

## Abstract

**BACKGROUND:**

In July 2012, the Japan Gastroenterological Endoscopy Society updated their guidelines for gastroenterological endoscopy in patients receiving antithrombotic therapy. Colonoscopic polypectomy procedures are associated with a high risk of bleeding.

**OBJECTIVES:**

The present study evaluated the safety of colonoscopic polypectomy procedures in terms of bleeding, among patients receiving antithrombotic therapy.

**DESIGN AND SETTING:**

Prospective observational study conducted in a tertiary-level public cardiovascular hospital in Istanbul, Turkey.

**METHODS:**

Colonoscopic polypectomies carried out in a single endoscopy unit between July 2018 and July 2019 were evaluated prospectively. The patients’ data, including age, gender, comorbidities, whether antithrombotic drug use was ceased or whether patients were switched to bridging therapy, polyp size, polyp type, polyp location, histopathology, resection methods (hot snare, cold snare or forceps) and complications relating to the procedures were recorded.

**RESULTS:**

The study was completed with 94 patients who underwent a total of 167 polypectomy procedures. As per the advice of the physicians who prescribed antithrombotic medications, 108 polypectomy procedures were performed on 60 patients without discontinuing medication and 59 polypectomy procedures were performed on 34 patients after discontinuing medication. The age, gender distribution and rate of bleeding did not differ significantly between the patients whose medication was discontinued and those whose medication was continued (P > 0.05).

**CONCLUSION:**

This study found that the colonoscopic polypectomy procedure without discontinuation of antithrombotic medication did not increase the risk of bleeding. This procedure can be safely performed by experienced endoscopists in patients with an international normalized ratio (INR) below 2.5.

## INTRODUCTION

Colonoscopic polypectomy is an effective treatment method that reduces the mortality associated with colorectal cancer,^1^ which is the second highest cause of cancer-related deaths. Colonoscopy as a screening test and colonoscopic polypectomy as an effective means of therapy reduce the risk of development of colon cancer by preventing progression of adenoma to carcinoma.^2^ As is the case with other interventional procedures, this procedure has been associated with some significant complications,^3^^,^^4^ among which postpolypectomy bleeding is the most common. The incidence of postpolypectomy bleeding ranges from 0.3% to 3.6% per patient.^5^^,^^6^ The use of antithrombotic medications is increasing worldwide, having been shown to be effective in preventing thrombosis. In conjunction with their increasing use by the general population, their use has also increased among patients undergoing endoscopic procedures.^7^

Colonoscopic diagnostic and therapeutic procedures need to be carried out with full awareness of the risk of hemorrhage.^8^ Discontinuation of antithrombotic medications prior to colonoscopic examination is believed to reduce the risk of gastrointestinal hemorrhage.^9^ However, the potential for thrombosis following discontinuation of antithrombotic medication is closely associated with major complications, including a high mortality rate.^10^ In 2012, the Japan Gastroenterological Endoscopy Society (JGES) published new guidelines regarding the use of anticoagulant and antiplatelet agents in endoscopic procedures.^11^ Colonoscopic polypectomy, among other endoscopic procedures, was described as a procedure presenting high risk of hemorrhage.

## OBJECTIVE

There are only a limited number of studies in the literature detailing polypectomy and its complications among patients receiving antithrombotic medication. The aim of the present study was to evaluate the safety of the colonoscopic polypectomy procedure among patients receiving antithrombotic medication.

## METHODS

In this observational study, colonoscopic polypectomies performed in a single center between July 2018 and July 2019 were evaluated prospectively. The study included patients aged 18 years and over who were on antithrombotic medication to treat cardiovascular complaints. The study primarily included patients undergoing diagnostic and therapeutic colonoscopic polypectomy due to suspected malignancy, to screen for malignancy, and investigations of the etiology of occurrences of anemia. Those undergoing hemostatic emergency procedures performed due to gastrointestinal hemorrhage, and those undergoing diagnostic endoscopic mucosal biopsies, were excluded. Colonoscopy procedures that were not completed due to inadequate bowel preparation and looping were also excluded. Lastly, only patients providing consent to participate in the study were included in the study.

Cessations of antithrombotic medication prior to endoscopic procedures, switches to bridging therapy and the continuation of these therapies during the procedure were decided upon by the cardiology physicians who prescribed the medication initially.

The data gathered on the patients included age, gender, comorbidities (hypertension, antihypertensive drug use, diabetes mellitus and antihyperglycemic drug use), whether use of antithrombotic drugs were ceased or whether the patient was switched to bridging therapy, proton pump inhibitor (PPI) use, polyp size, polyp type, polyp location, histopathology, characteristics of the surgical margin, resection methods (hot snare, cold snare or forceps) and complications relating to the procedures. In the present study, the anticoagulant medications included warfarin (5 mg) and rivaroxaban (15 mg), and the antiplatelet medications included aspirin (100 mg) and clopidogrel (75 mg). Because of the single-center study design, no other anticoagulant or antiplatelet medications were included due to the limited number of cases.

No interventions were made if the prothrombin time (PT-INR) level was above 2.5 in patients undergoing warfarin therapy, given that the risk of hemorrhage was significantly higher in such patients. If the international normalized ratio (INR) was above 2.5, the prescribing physician was consulted, and either warfarin therapy was discontinued or the patient was switched to bridging therapy to reduce their INR to below 2.5 prior to the procedure.

The polyp size was recorded as the actual size in millimeters, measured histopathologically. Polyp type was classified as sessile, pedunculated or flat, in accordance with the Paris classification guidelines.^12^ Polyp location was defined as either in the proximal colon (cecum, ascending colon and transverse colon) or in the distal colon (descending colon, sigmoid colon and rectum). The histopathological classification included carcinoma, high-grade dysplasia, moderate dysplasia, low-grade dysplasia, tubular adenoma, serrated adenoma, hyperplastic polyp, inflammatory polyp or missing.

The colonoscopies were performed by endoscopists in a single center. Polypectomies were performed using cold or hot snares (Micro-Tech, Nanjing, China) or forceps (Micro-Tech, Nanjing, China; 2.3 mm). The method to be used was decided upon by the operating endoscopist during the procedure. Coagulation catheters were used in the event of bleeding. All procedures were performed using the same endoscopy system.

The patients were advised to return to the clinic if they experienced hematemesis or melena after the polypectomy and were invited to attend a control visit four weeks after the polypectomy. Postpolypectomy bleeding (PPB) was defined as bleeding occurring within four weeks of the polypectomy. A diagnosis of PPB was established by means of emergency colonoscopy in patients presenting with hematochezia, based on the presence of active bleeding, a newly formed blood clot or visible vessels at the polypectomy site, and on the presence of intraluminal blood. PPB was ruled out if the hematochezia was mild and self-limiting without requiring emergency colonoscopy.^13^

This study was approved by the ethics committee of our hospital (approval no. 2018.4/1-98; approval date: May 25, 2018).

### Statistical analysis

Descriptive statistics were presented as numbers and percentages for categorical variables, while quantitative variables were presented as mean, standard deviation, minimum, maximum and median values. The normality of the distribution of the data was ascertained using the Kolmogorov-Smirnov test. A Mann-Whitney U-test was used for the analysis on quantitative independent variables. A chi-square test was used in the analysis on qualitative variables and Fisher’s exact test was used if the conditions were not met for a chi-square test. The SPSS 22.0 for software package (IBM Corp, Armonk, NY, United States) was used for the statistical analysis.

## RESULTS

**Figure 1.**
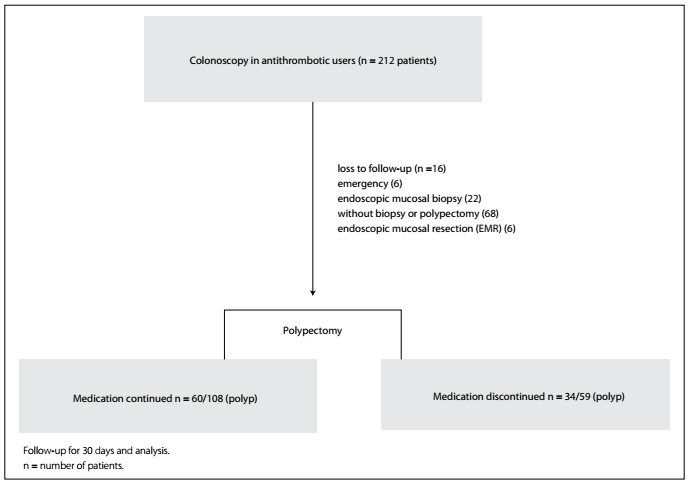
Flow chart of the present study: antithrombotic users.

The study was completed with 94 patients who underwent colonoscopic polypectomy. A total of 167 polypectomy procedures were performed on these 94 patients (Figure 1). The general characteristics and demographic data of the patients is presented in Table 1.

**Table 1.** Demographic data

		Min-Max	Median	Median ± SD or n (%)
Age		44.0-83.0	65.0	65.3 ± 8.4
Sex	Female			29 (30.9%)
	Male			65 (69.1%)
Indication	Malignancy screening			45 (47.9%)
	Anemia			49 (52.1%)
Disease	Atrial fibrillation			9 (9.6%)
	Coronary bypass			6 (6.4%)
	CAD			31 (33.0%)
	Mitral valve replacement			8 (8.5%)
	Cardiac stents			36 (38.3%)
	Cardiac insufficiency			4 (4.3%)
Antithrombotic	Aspirin (100 mg)			42 (44.7%)
	Warfarin			16 (17.0%)
	Clopidogrel			24 (25.5%)
	Clopidogrel+ aspirin			2 (2.1%)
	Rivaroxaban			10 (10.6%)
LMWH bridging	No			68 (72.3%)
	Yes			26 (27.7%)
Diabetes mellitus	No			69 (73.4%)
	Yes			25 (26.6%)
Hypertension	No			45 (47.9%)
	Yes			49 (52.1%)
CAD	No			63 (67.0%)
	Yes			31 (33.0%)
Bleeding	No			92 (97.8%)
	Yes			2 (2.2%)
Platelets		126.00-495.00	231.50	241.26 ± 72.07
INR		0.88-2.10	1.04	1.13 ± 0.25
Continued medication	Yes			60 (63.8%)
	No			34 (36.2%)
PPI	Yes			48 (51.1%)
	No			46 (48.9%)
Polyp type	Flat			5 (3.0%)
	Pedunculated			17 (10.2%)
	Sessile			145 (86.8%)
Location	Distal colon			100 (59.9%)
	Proximal colon			67 (40.1%)
Polyp size (mm)		4.0-20.0	7.0	7.9 ± 3.3
Pathology				
Inflammatory polyp				2 (1.2%)
High-grade dysplasia				4 (2.4%)
Hyperplastic polyp				41 (24.6%)
Intramucosal adenocarcinoma				1 (0.6%)
Lost pathology				2 (1.2%)
Low-grade dysplasia				50 (29.9%)
Moderate dysplasia				6 (3.6%)
Serrated polyp				5 (3.0%)
Tubular adenoma				56 (33.5%)

Min-Max = minimum-maximum; SD = standard deviation; CAD = coronary artery disease; LMWH = low molecular weight heparin; INR = international normalized ratio; PPI = proton pump inhibitor.

As per the advice of the physicians who prescribed the antithrombotic medications, 108 polypectomy procedures were performed on 60 patients whose medication was continued, and 59 polypectomy procedures were performed on 34 patients whose medication was discontinued. Among the 34 patients whose medication was discontinued, 26 were switched to bridging therapy with low-molecular-weight heparin (LMWH).

The age, gender distribution and rate of bleeding did not differ significantly between those whose medication was discontinued and those whose medication was continued (P > 0.05). The rate of use of bridging therapy was significantly higher among the patients whose medication was discontinued than among those whose medication was continued (P < 0.05). The INR and platelet count were significantly higher in those whose medication was discontinued than in those whose medication was continued (P < 0.05) (Table 2).

**Table 2.** Groups that discontinued and continued the drug during polypectomy

		Medication continued		Medication discontinued		P
		Median ± SD or n (%)	Median	Median ± SD or n (%)	Median	
Age		64.2 ± 9.0	62.5	64.5 ± 5.8	64.5	0.741^m^
Sex	Female	20 (33.3%)		9 (26.5%)		0.489^χ²^
	Male	40 (66.7%)		25 (73.5%)		
Indication	Malignancy screening	31 (51.7%)		14 (41.2%)		0.328^χ²^
	Anemia	29 (48.3%)		20 (58.8%)		
Disease	Atrial fibrillation	2 (3.3%)		7 (20.6%)		**0.006**^χ²^
	Coronary bypass	4 (6.7%)		2 (5.9%)		0.881^χ²^
	CAD	26 (43.3%)		5 (14.7%)		**0.005**^χ²^
	Mitral valve replacement	3 (5.0%)		5 (14.7%)		0.105^χ²^
	Cardiac stents	23 (38.3%)		13 (38.2%)		0.993^χ²^
	Cardiac insufficiency	2 (3.3%)		2 (5.9%)		0.618^χ²^
Antithrombotic	Aspirin (100 mg)	33 (55.0%)		9 (26.5%)		**0.008**^χ²^
	Warfarin	4 (6.7%)		12 (35.3%)		**0.000**^χ²^
	Clopidogrel	13 (21.7%)		11 (32.4%)		0.254^χ²^
	Clopidogrel + aspirin	2 (3.3%)		0 (0.0%)		0.533^χ²^
	Rivaroxaban	8 (13.3%)		2 (5.9%)		0.260^χ²^
LMWH bridging	No	60 (100.0%)		8 (23.5%)		**0.000**^χ²^
	Yes	0 (0.0%)		26 (76.5%)		
Diabetes mellitus	No	46 (76.7%)		23 (67.6%)		0.342^χ²^
	Yes	14 (23.3%)		11 (32.4%)		
Hypertension	No	32 (53.3%)		13 (38.2%)		0.159^χ²^
	Yes	28 (46.7%)		21 (61.8%)		
Bleeding	No	58 (96.7%)		33 (97.1%)		1.000^χ²^
	Yes	1 (1.7%)		1 (2.9%)		
Platelets		231.8 ± 64.1	225.0	249.2 ± 54.5	250.0	**0.040**^m^
INR		1.1 ± 0.2	1.0	1.2 ± 0.3	1.1	**0.009**^m^

^m^Mann-Whitney U test; ^χ²^chi-square test. SD = standard deviation; CAD = coronary artery disease; LMWH = low molecular weight heparin; INR = international normalized ratio.

The distribution of localization did not differ significantly between the forceps and snare groups (P > 0.05). Polyp size was significantly greater in the snare group than in the forceps group (P < 0.05) (Table 3). The distribution of polyp type did not differ significantly between the cold snare and hot snare groups (P > 0.05). The distribution of localization did not differ significantly between the cold snare and hot snare groups (P > 0.05). Polyp size was significantly greater in the hot snare group than in the cold snare group (P < 0.05) (Table 4).

**Table 3.** Types of polypectomy

		Forceps		Snare		P
		Median ± SD or n (%)	Median	Median ± SD or n (%)	Median	
Polyp type	Flat	0 (0.0%)		5 (5.5%)		**0.004**^χ²^
	Pedunculated	3 (3.9%)		14 (15.4%)		
	Sessile	73 (96.1%)		72 (79.1%)		
Location	Distal colon	40 (52.6%)		60 (65.9%)		0.081^χ²^
	Proximal colon	36 (47.4%)		31 (34.1%)		
Polyp size (mm)		4.8 ± 1.0	5.0	7.8 ± 3.2	7.0	**0.000**^m^

^m^Mann-Whitney U test; ^χ²^chi-square test. SD = standard deviation.

**Table 4.** Types of snare polypectomy

		Cold				Hot				P
		Median ± SD or n %			Median	Median ± SD or n %			Median	
Polyp type	Flat	1		2.1%		4		10.3%		0.054^χ²^
	Pedunculated	5		10.4%		9		23.1%		
	Sessile	42		87.5%		26		66.7%		
Location	Distal colon	32		66.7%		26		66.7%		1.000^χ²^
	Proximal colon	16		33.3%		13		33.3%		
Polyp size (mm)		7.0	±	2.1	6.0	9.0	±	4.0	8.0	**0.011**^m^

^m^Mann-Whitney U test; ^χ²^chi-square test. SD = standard deviation.

Postpolypectomy bleeding occurred in two patients: one among the patients who used aspirin and whose medication was not discontinued; and one among the patients who used clopidogrel and whose medication was discontinued (Table 5). No thromboembolic events occurred in any of the participating patients.

**Table 5.** Bleeding patients

	Patient 1	Patient 2
Age	58	71
Sex	Female	Male
Drug	Aspirin (100 mg)	Clopidogrel
Disease	Coronary bypass	Cardiac stents
Location	Distal colon	Distal colon
Polyp size	8 mm	11 mm
Indication	Malignancy screening	Malignancy screening
Resection type	Forceps	Hot snare
With cessation	No	Yes
INR	0.96	1.04

INR = international normalized ratio.

## DISCUSSION

The present single-center, prospective and observational study investigated the risk of bleeding following colonoscopic polypectomy procedures performed with or without cessation of medication, among patients who had been receiving antithrombotic medication. The results from the study suggest that colonoscopic polypectomy procedures without discontinuation of drugs among patients who had been receiving antithrombotic medication did not increase the risk of bleeding within 30 days of the procedure.

Aspirin is in common use for secondary prophylaxis against cardiovascular events and increases the bleeding time following endoscopic biopsy and polypectomy.^14^ In a retrospective cohort study on 450 polypectomy procedures performed on 145 patients, comparing those who received aspirin with those who did not receive aspirin, Pan et al.^15^ detected postpolypectomy bleeding in eight of the 145 patients, with a significantly higher risk of bleeding, compared with those who did not use aspirin.

According to the 2009 guidelines published by the American Society for Gastrointestinal Endoscopy (ASGE), cessation of aspirin usage is recommended in gastroenterological endoscopic procedures in which there is a high risk of bleeding.^16^ Similarly, the guidelines of the European Society of Gastrointestinal Endoscopy (ESGE) recommend that aspirin therapy should be continued but then ceased five days prior to the procedure, among patients at low risk of thromboembolic events.^17^ In the 2012 guidelines of the Japan Gastroenterological Endoscopy Society (JGES), cessation of aspirin therapy prior to a colonoscopic polypectomy procedure was not recommended.^11^

In a retrospective study, Yousfi et al.^18^ showed that aspirin use did not increase the risk of bleeding; and a similar study by Manocha et al.^19^ showed that aspirin use does not increase the risk of bleeding (3% versus 3.2%). In the present study, 73 polypectomy procedures were performed on 42 patients who were on aspirin therapy, and bleeding occurred in one patient. This bleeding rate was considered acceptable and comparable to the rates reported in the literature. We consider that the procedure may be performed safely in experienced clinics without cessation of this drug, considering the possibility that serious thromboembolic events could occur in cases of discontinuation of aspirin use.

Clopidogrel is a strong inhibitor of platelet adhesion and aggregation, and its use has increased significantly worldwide. The number of colonoscopic procedures performed while receiving this drug has also increased in parallel with the overall increase in the frequency and duration of clopidogrel use. In a meta-analysis on five studies, Gandhi et al.^20^ reported that bleeding occurred following colonoscopic polypectomy in 37 (6.45%) out of 574 patients who used clopidogrel and that the risk of bleeding was significantly higher than in the control group. In contrast to this study, Chan et al.^21^ reported that there was no significant difference in postpolypectomy bleeding in a randomized controlled study on 449 polypectomy procedures performed on 216 patients. Likewise, Singh et al.^22^ showed in a retrospective study that clopidogrel use alone did not increase the risk of bleeding. In the present study, 39 polypectomy procedures were performed on 24 patients who were on clopidogrel therapy. The procedure was performed without cessation of the drug in 13 patients and with cessation of the drug in 11 patients. Bleeding occurred following the procedure in one patient whose medication was discontinued. Although no bleeding occurred in any patient in the group that continued to use the medication, these patients required close monitoring after the procedure, due to the higher risk of bleeding than with other antiplatelet medications.

The main guidelines recommend cessation of anticoagulants and bridging heparin therapy prior to polypectomy among patients at high risk of thrombosis.^11^^,^^23^^,^^24^ In a retrospective study by Kubo et al.,^25^ anticoagulant use was shown to be an independent risk factor, even if the anticoagulant medication was discontinued and the patient was switched to bridging therapy before the procedure. There have been many studies showing that a relationship exists between anticoagulant use and postpolypectomy bleeding.^26^^,^^27^ Similarly, Sakai et al.^28^ compared the bridging treatment group and the control group that did not use anticoagulants and found that there was higher risk of bleeding in the group treated with bridging. In the present study, 34 polypectomy procedures were performed on 16 patients who were on warfarin therapy. The polypectomy was performed without cessation of warfarin in four patients, whereas 12 patients were switched to bridging therapy prior to the procedure. No bleeding occurred during these procedures. Anticoagulant use and polypectomy procedures are still classified as high-risk procedures in the current guidelines. We suggest that utmost attention should be paid to the risk of bleeding, even if the international normalized ratio has returned to normal ranges.

In comparison with bleeding complications, thromboembolic events may have more serious consequences in patients whose antithrombotic medication is discontinued prior to an endoscopic biopsy.^9^ Over recent years, systematic reviews and meta-analyses have been conducted to determine the risk of postpolypectomy bleeding in patients receiving antithrombotic therapy.^29^ In one meta-analysis, Pigò et al.^30^ showed that aspirin use increased the risk of bleeding from colorectal polyps. Similarly, Chan et al.,^21^ in a randomized controlled study, found that clopidogrel use did not increase the risk of bleeding during or after the procedure. In a study involving 210 patients, Kubo et al.^25^ found that there was no significant increase in the risk of bleeding other than with anticoagulant use and with bridging therapy in patients receiving antithrombotic medications. In a similar study on 906 patients, Ishigami et al.^13^ showed that anticoagulant use and bridging heparin therapy did not increase the risk of bleeding. In the present study, polypectomies were performed without cessation of antithrombotic medication in 60 out of 94 patients. Bleeding occurred following the procedure in one patient. In line with the current guidelines, we believe that polypectomies can be performed without discontinuing antiplatelet drugs such as aspirin and clopidogrel. Nonetheless, we recommend that patients on warfarin therapy should undergo the procedure with careful monitoring in well-established clinics, even if the international normalized ratio values are found to be within normal ranges.

The limitations of this study include its single-center design, the lack of sufficient randomization between the groups due to the observational nature of the study and the lack of power analysis, since the patients were using a wide variety of drugs. In addition, this study did not include patients with a high risk of bleeding, since the procedure was not performed on patients with an international normalized ratio level above 2.5. This was an important limitation of the study.

## CONCLUSION

The present study showed that colonoscopic polypectomy procedures without discontinuation of antithrombotic medications do not increase the risk of bleeding. High-risk procedures such as colonoscopic polypectomies can be safely performed by experienced endoscopists in patients with an international normalized ratio below 2.5. However, these procedures are not necessarily mandatory in any patient undergoing anticoagulation.
